# Exploring the Physiological Effects and Mechanisms of Gamma-Aminobutyric Acid in Food Systems

**DOI:** 10.3390/foods15183323

**Published:** 2026-09-19

**Authors:** Mingcan Liu, Shuyun Zhu, Tabussam Tufail, Bin Xu

**Affiliations:** School of Food Science and Engineering, Jiangsu University, Zhenjiang 212013, China; 19811711926@163.com (M.L.); shyzhu@ujs.edu.cn (S.Z.); tabussam.qadri@gmail.com (T.T.)

**Keywords:** GABA, antihypertensive mechanism, anxiolytic effect, neurotransmitter regulation, functional foods

## Abstract

Gamma-aminobutyric acid (GABA), a non-protein amino acid widely present in plant-based foods, has gained considerable attention for its diverse physiological effects and underlying mechanisms. As a functional dietary component, GABA has been reported to exhibit antihypertensive activity in preclinical and preliminary human studies, with proposed mechanisms including angiotensin-converting enzyme (ACE) inhibition and modulation of sympathetic outflow. In nematode models, GABA has been observed to alleviate aging-related oxidative stress by enhancing antioxidant enzymes (e.g., SOD, CAT) and reducing lipofuscin accumulation. It may also alleviate certain menopausal symptoms based on limited human trial data, potentially through regulation of autonomic nervous system balance and reduction in vasomotor disturbances. Its anxiolytic and sleep-promoting effects are linked to GABAergic neurotransmission via GABAA/B receptors, which suppresses hypothalamic–pituitary–adrenal (HPA) axis hyperactivity. GABA also shows anticancer potential in cell-based assays by disrupting tumor cell cycles and metastasis pathways, while its antidiabetic role in preclinical models involves pancreatic β-cell protection and glucose homeostasis regulation. Despite the wide range of proposed physiological benefits, safety assessments indicate that GABA is generally well-tolerated at typical dietary supplement doses (≤300 mg/day), with higher intakes (up to 3000 mg/day) permitted in some regulatory frameworks, supporting its use in functional foods. Future research should focus on optimizing GABA-enriched food production and elucidating dose-related mechanisms for personalized nutrition.

## 1. Introduction

A small amount of the functional factor, gamma-aminobutyric acid (abbreviated as GABA), is present in mature plant seeds, fruit pulp, fermented foods, and so on. The molecular formula of GABA is NH_2_(CH_2_)_3_COOH, its molecular mass is 103.12 g/mol, and its melting point is 202 °C [1,2], and it has good thermal stability. Its appearance is usually a white or light yellow crystalline solid, and it dissolves easily in water but is difficult to dissolve or is insoluble in various organic solvents [1,3].

GABA is a non-protein amino acid widely distributed in nature. It is synthesized by the decarboxylation of glutamic acid. It was found in potato tubers, green tea, soybeans, germinated brown rice, and other plant foods. So, it was only regarded as a metabolite in plants and microorganisms at the beginning [4,5]. However, GABA was later also found in animal brains, where it acts mainly as an inhibitory neurotransmitter in the central nervous system, including the brain and spinal cord. [6,7]. In addition, GABA content in pancreatic β cells and intestinal muscle interlamellar nerve plexus cells is also comparable to that in the brain [8,9]. In humans, GABA is packaged into synaptic vesicles via the vesicular GABA transporter (VGAT), later released in the synaptic cleft, and then binds to the GABA receptors in the postsynaptic region, which are either GABA_A_ or GABA_B_ receptors [10]. GABA_A_ receptors are heteropentameric ligand-gated chloride channels. GABA_A_ receptor activation increases chloride conductance, hyperpolarizes the postsynaptic membrane, and dampens neural firing. Studies in rodents have implicated GABA_A_ receptors in the central regulation of blood pressure, anxiety, and seizure susceptibility. These findings reflect that GABA_A_ receptors are the principal mediators of fast synaptic inhibition throughout the mammalian central nervous system, with functions extending to sensory processing, motor coordination, and cognitive modulation [10]. Upon activation, GABA_B_ receptors couple to inwardly rectifying potassium channels (GIRKs), inducing slow and prolonged hyperpolarization; presynaptic GABA_B_ receptors inhibit voltage-gated calcium channels, thereby reducing neurotransmitter release from the terminal. This dual pre- and postsynaptic inhibitory mechanism gives GABA_B_ receptors a powerful modulatory influence over synaptic transmission and network excitability. Rodent and human pharmacological studies have suggested their involvement in pain modulation, emotional regulation, memory consolidation, motor learning, and neuroprotection [11,12].

The growing awareness of health and wellness among the public, coupled with the reported biological activity of gamma-aminobutyric acid (GABA), has led to an increased focus on the development of GABA-rich foods. As a result, GABA has become a popular ingredient in many dietary supplements (DS). Industry projections indicate that the global market for GABA dietary supplements is expected to rise significantly, from 38 million dollars in 2019 to 50 million dollars by the end of 2026. The main purpose of this study is to review the mechanisms, physiological functions, pharmacological actions, and safety of GABA in humans. This comprehensive review aims to provide valuable theoretical insights and references to support the ongoing research and the application of GABA in functional foods and supplements.

## 2. Natural Occurrence and Dietary Sources of GABA

GABA is primarily obtained through the consumption of plant-based foods in the daily diet, including a variety of grains, fruits, and vegetables [2,13]. However, the natural GABA content in plants is typically low, with only specific elite varieties exhibiting higher concentrations of GABA [14]. For example, the GABA content in grains ranges from 0.67 to 54.00 mg/100 g, with brown rice and barley having the highest GABA contents of 27.00 mg/100 g and 54.00 mg/100 g, respectively. (The germ and bran of rice are rich in GABA; the GABA content of barley bran can reach 350 mg/100 g) [15,16]. Due to the removal of the rice germ, the GABA content of refined white rice for daily consumption is only 0.5–1.2 mg/100 g. Among fruits and vegetables, lychee, tomato, spinach, and some other foods are notable for their high GABA content [17,18], although most GABA contents are less than 34 mg/100 g.

Due to the deficient GABA content of most plant foods, human health needs cannot be met when relying only on a daily diet. As shown in Table 1, GABA levels varied widely across food categories. Although the GABA content of foods such as lychee, tomato, and spinach can exceed 200 mg/100 g, people cannot consume such foods in large quantities daily [2]. For example, 100 g of soy sauce contains nearly 70 mg GABA, which is five times that of germinated brown rice. Consequently, the daily intake of soy sauce per person is usually 1–2 g, so the maximum daily intake of GABA is 1.4 mg [19]. As a result, GABA is commonly used as an additive in the production of GABA-rich functional foods. However, the physiological effects of GABA-enriched or fermented foods depend on the administered dose, intervention duration, bioavailability, and accompanying food components [20]. GABA-rich foods should be considered promising functional-food vehicles, whereas their anxiolytic, antidepressant, sleep-promoting, antihypertensive, anti-aging, anticancer, and antidiabetic effects should be described as preliminary or evidence-dependent rather than established clinical benefits in humans [21].

## 3. Potential Role of Dietary GABA in Blood Pressure Regulation

Evidence from short-term human intervention trials and hypertensive animal models suggests that dietary or supplemental GABA may produce a modest blood-pressure-lowering effect, particularly in individuals with high-normal, borderline, or mild hypertension. However, the evidence is not equivalent across experimental models [65,66,67]. Human studies have generally been small and short in duration and have used heterogeneous food matrices, including fermented milk, GABA-enriched chlorella, and GABA-enriched rice. Mechanistic evidence has mainly been obtained from rodents, isolated tissues, or in vitro assays. The most consistent human finding concerns systolic blood pressure (SBP), whereas effects on diastolic blood pressure (DBP) are less consistent [68,69]. Therefore, GABA-containing foods should be regarded only as potential adjuncts to established lifestyle and pharmacological interventions, not as substitutes for antihypertensive treatment.

### 3.1. Evidence from Preclinical and Human Studies

#### 3.1.1. Evidence from Hypertensive Animal Models

Preclinical studies have demonstrated that GABA administration attenuates blood pressure elevation in rodent models of induced hypertension, whereas no significant effect is observed in normotensive controls. In one clinical investigation, individuals with high-normal blood pressure consumed 100 mL of fermented milk containing 12.3 mg GABA daily. After eight weeks, SBP decreased after eight weeks, and diastolic blood pressure (DBP) followed suit after 12 weeks [70,71]. Cessation of the GABA-fortified milk, however, resulted in a return of blood pressure to baseline within four weeks. A fermented GABA-salt preparation produced a similar pattern in a diet-induced hypertensive mouse model. Treatment was associated with lower blood pressure and attenuation of pro-inflammatory macrophage polarization, endothelial dysfunction, and vascular smooth muscle proliferation. These changes suggest that peripheral vascular and inflammatory mechanisms may contribute to the observed antihypertensive effects. Other rodent studies have implicated the autonomic and angiotensin-receptor pathways [68,69]. The responses observed in rodents therefore generate mechanistic hypotheses but cannot establish the magnitude or mechanism of an effect in humans. Human intervention studies should be considered separately and should compare purified GABA with composition-matched GABA-containing foods.

#### 3.1.2. Evidence from Controlled Human Intervention Studies

A study reported that subjects taking 4 g of GABA-rich chlorella daily could reduce blood pressure in hypertensive patients. In the subjects with high-normal blood pressure, compared to baseline. The SBP decreased significantly after taking GABA-rich chlorella at the 8th to 14th weeks, compared with no significant decrease in systolic blood pressure in the placebo group [72]. The DBP also decreased after taking GABA-rich chlorella. In the subjects with borderline hypertension (blood pressure values between normal blood pressure and confirmed hypertension), compared with baseline, taking GABA-rich chlorella showed a significant reduction in systolic blood pressure at the 4th to 12th and 16th weeks [68]. Diastolic blood pressure in patients with borderline hypertension was significantly lower than baseline at the 4th to 12th, and 16th weeks [70,71]. No significant change in DBP was observed within the placebo group, while DBP values in the placebo group remained consistently higher than those in the GABA-treated groups at all measured time points. However, chlorella contains proteins, minerals, pigments, and other bioactive constituents, the response cannot be attributed solely to GABA. In another randomized, double-blind trial, 39 adults with mild hypertension consumed 150 g/day of GABA-enriched white rice containing 11.2 mg of GABA per 100 g, compared with lower-GABA rice containing 2.7 mg of GABA per 100 g, for 8 weeks. Morning home blood pressure was reduced at specific time points, although the pattern was not consistent across all measurements. This pattern suggests a possible time- and measurement-dependent effect, but the low-GABA rice comparator does not completely isolate the contribution of GABA.

An earlier intervention in adults with high-normal blood pressure used 100 mL/day of fermented milk containing approximately 12.3 mg of GABA. Systolic blood pressure decreased after the 8th week and diastolic blood pressure after 12 weeks, but blood pressure returned toward baseline within 4 weeks of discontinuation [73]. Across these and similar short-term trials, sample sizes were small, intervention periods were brief (8–12 weeks), and GABA doses, food matrices, control interventions, and blood-pressure measurement schedules varied considerably. The overall signal is more consistent for SBP—particularly morning or home measurements—than for diastolic blood pressure. Collectively, these findings support a possible modest short-term effect on systolic or morning blood pressure in some individuals with high-normal blood pressure, borderline hypertension, or mild hypertension, but they do not establish efficacy for established or pharmacologically treated hypertension. GABA-containing foods may therefore be investigated as dietary adjuncts, not substitutes for standard antihypertensive therapy.

### 3.2. Mechanistic Pathways of GABA-Mediated Blood Pressure Regulation

#### 3.2.1. Central Modulation via At_2_ Receptor-Dependent Gabaergic Signaling

The central antihypertensive action of GABA has been primarily mapped in rodent brainstem preparations using site-specific microinjections, receptor-selective agonists/antagonists, and electrophysiological recordings. It is essential to recognize that this model describes what happens when GABA is delivered directly into specific brain nuclei, not what necessarily occurs after oral consumption of GABA-containing foods.

**Figure 1:**
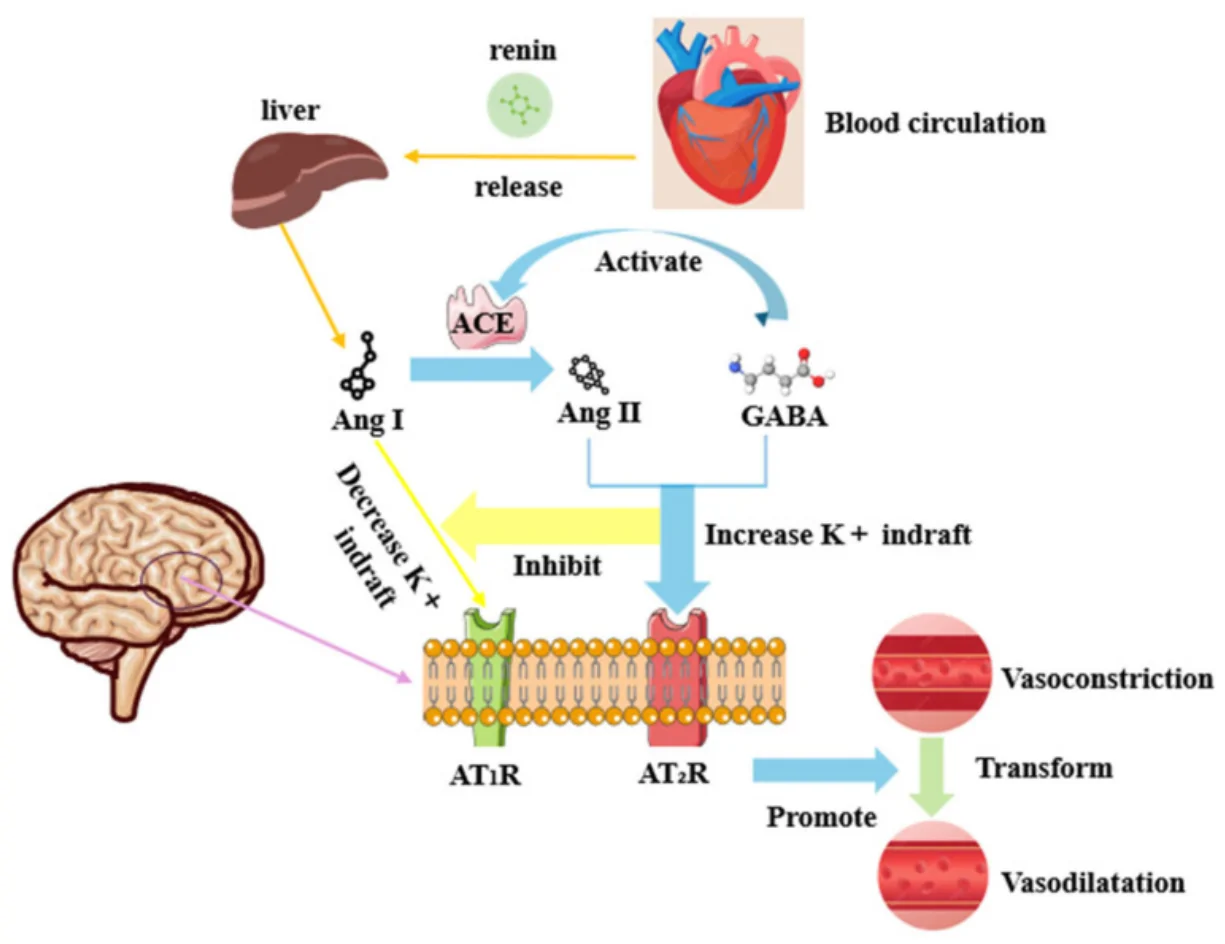
Mechanistic pathways of GABA-mediated blood pressure regulation via the renin–angiotensin system.

**Figure 2:**
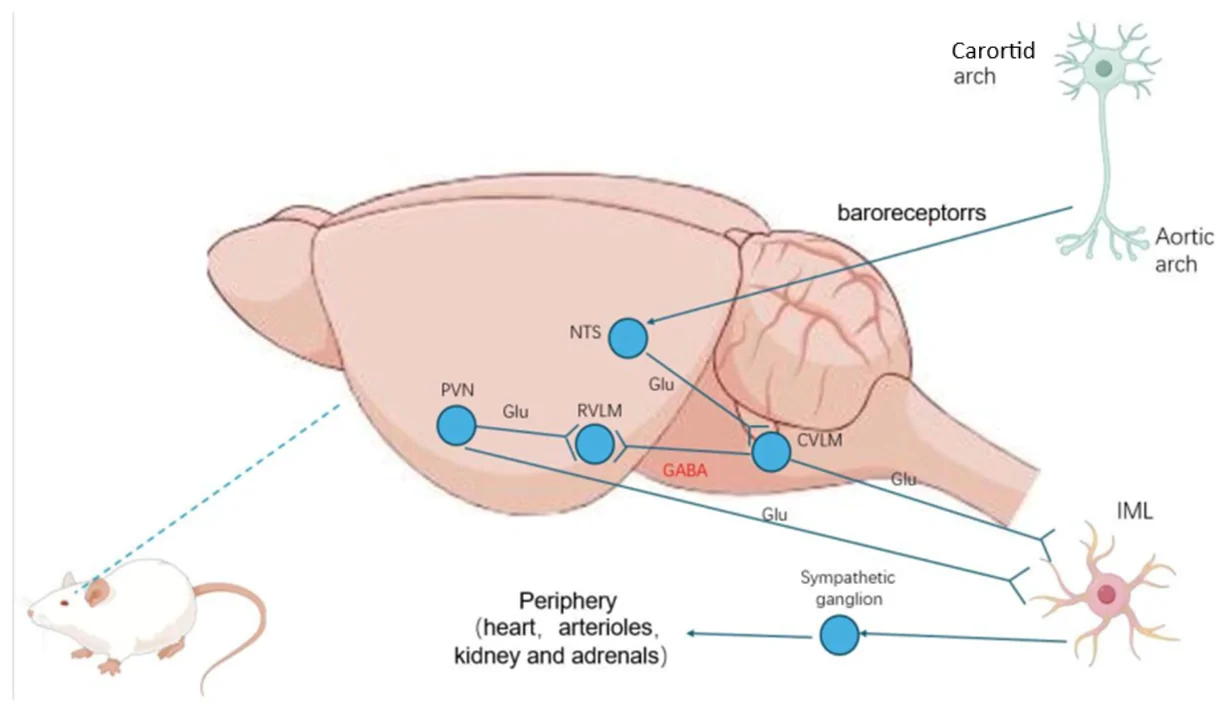
Sagittal section of the rat brain showing the GABAergic and glutamatergic projections in the blood pressure-regulating nuclei. **Notes:** glutamate (Glu), gamma-aminobutyric acid (GABA), nucleus tractus solitarius (NTS), caudal ventrolateral medulla (CVLM), rostral ventrolateral medulla (RVLM), paraventricular nucleus (PVN), and intermediolateral (IML) column.

As shown in Figure 1 and Figure 2, rodent studies have suggested that GABA modulates blood pressure through complex interactions among several brainstem nuclei—including the paraventricular nucleus of the hypothalamus (PVN), the nucleus tractus solitarius (NTS), the caudal ventrolateral medulla (CVLM), and the rostral ventrolateral medulla (RVLM)—and their associated angiotensin II receptor subtypes [72]. In this model, the NTS is densely populated with angiotensin II type 2 receptors (AT_2_R), which are predominantly located on inhibitory GABAergic neurons. Activation of AT_2_R in the RVLM elicits hypotensive responses via a GABA-dependent mechanism [74].

Mechanistically, the rodent model describes the following sequence: AT_1_ receptor activation decreases neuronal K^+^ channel currents and augments the firing rate of RVLM presympathetic neurons, promoting vasoconstriction and hypertension. Conversely, AT_2_ receptor activation increases K^+^ channel currents and dampens RVLM neuronal excitability, counterbalancing AT_1_-mediated effects and favoring vasodilation [75]. A related pathway involves GABAergic neurons in the NTS that release nitric oxide (NO). This NO then acts on presynaptic terminals of RVLM neurons to stimulate AT_2_R on RVLM GABAergic neurons. This facilitates GABA release from various brain regions, reducing sympathetic outflow and ultimately lowering blood pressure. Experimental support for this model comes from rodent studies demonstrating that administration of the AT_2_R agonist C21 significantly reduces blood pressure—an effect that was abolished by the AT_2_R antagonist PD123319 [75].

Therefore, we interpret the central GABAergic pathway as a well-established neurophysiological mechanism in animal models that provides a plausible biological rationale for antihypertensive effects, but whose actual engagement by orally consumed dietary GABA in humans remains unproven. This is not to dismiss the pathway, but to calibrate its evidential weight appropriately.

#### 3.2.2. Interaction of GABA with the Renin–Angiotensin System

**Figure 3:**
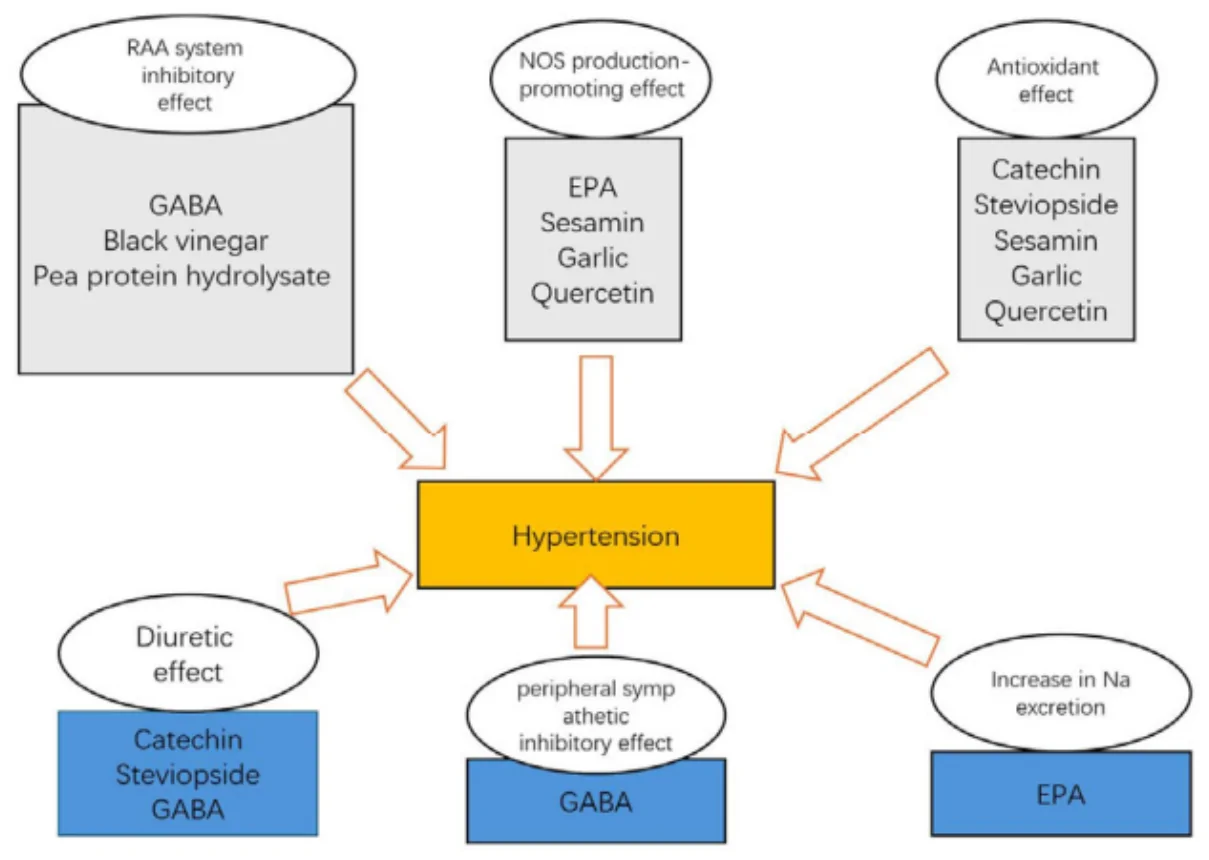
Antihypertensive mechanism of functional foods. **Note:** RAAS: Rennin-angiotensin-aldosterone.

As shown in Figure 3, both peripheral and central renin–angiotensin systems (RAS) are pivotal in blood pressure homeostasis, and many clinical antihypertensive agents act through ACE inhibition. The classical RAS pathway begins with renin-mediated cleavage of angiotensinogen to angiotensin I (Ang I). ACE subsequently transforms Ang I into biologically active angiotensin II (Ang II). Elevated Ang II stimulates aldosterone secretion, promoting renal sodium reabsorption and plasma volume expansion, while simultaneously inducing vasoconstriction and sympathetic activation—effects that collectively elevate arterial pressure.

In vitro assays using rat-derived ACE have demonstrated that GABA, along with alanine, theanine, and γ-hydroxybutyric acid, substantially inhibits enzymatic activity, with GABA exhibiting the most potent inhibitory effect [76,77]. By curtailing ACE function, GABA would theoretically limit the conversion of Ang I to Ang II, thereby reducing circulating Ang II levels and lowering blood pressure [78]. However, these in vitro assays typically use GABA concentrations in the millimolar range, whereas peak plasma concentrations after oral supplementation are in the low micromolar range—a difference of approximately two to three orders of magnitude. This concentration gap raises substantial questions about the physiological relevance of ACE inhibition as a primary mechanism for GABA’s blood-pressure-lowering effect in humans. Thus, while the ACE-inhibitory pathway is well documented in biochemical assays, its relative contribution to the overall hypotensive effect of GABA in humans remains unclear. Animal studies suggest that alternative mechanisms—such as direct modulation of sympathetic tone or vascular smooth muscle relaxation—may play equally important roles. Moreover, most human trials have focused on hemodynamic endpoints without rigorously dissecting the underlying neurohumoral pathways. Future research should therefore employ selective receptor antagonists and genetic models to delineate the relative contributions of central GABAergic signaling versus peripheral RAS inhibition, particularly in elderly or hypertensive populations where polypharmacy may raise safety concerns.

Beyond central modulation, GABA exerts direct peripheral effects by interfering with the renin–angiotensin system (RAS), a key hormonal pathway that governs blood pressure homeostasis. The underlying mechanisms can be summarized as follows: (i) GABA acts as a competitive inhibitor of angiotensin-converting enzyme (ACE), as demonstrated in in vitro assays using rat-derived ACE, where GABA exhibits more potent inhibitory activity than alanine, theanine, or γ-hydroxybutyric acid. (ii) By reducing ACE activity, GABA limits the conversion of angiotensin I to angiotensin II (Ang II), thereby lowering circulating Ang II levels. (iii) The diminished Ang II leads to decreased aldosterone secretion, reduced renal sodium reabsorption, and attenuated vasoconstriction, collectively lowering arterial pressure [79,80].

While animal models have not fully supported the canonical pathway of angiotensin-converting enzyme inhibition, they point to alternative mechanisms whereby GABA may exert hypotensive effects—either through central or peripheral modulation of sympathetic tone, or via direct influences on vascular smooth muscle function. In contrast, human investigations to date have predominantly centered on hemodynamic endpoints, leaving critical questions regarding underlying neurohumoral mechanisms, receptor subtype specificity, and long-term safety profiles unresolved [7,81]. These knowledge gaps are especially pertinent among elderly individuals and patients with coexisting cardiovascular conditions. Therefore, the potential synergies, optimal dosing parameters, and adverse effect profiles of combining GABA with the standard antihypertensive regimens require rigorous evaluation through large-scale, meticulously designed clinical trials.

## 4. The Anti-Aging Role of GABA

### 4.1. Mitigation of Aging Biomarkers by GABA

Current evidence for GABA-related anti-aging effects is predominantly preclinical and should not be equated with evidence of clinical anti-aging efficacy. Tu et al. sprayed mulberry leaves with 0.086–8.6 mM GABA and fed them to silkworms. GABA exposure was associated with increased adult fecundity, higher glutathione content, and higher superoxide dismutase/catalase activities, as well as a higher NAD^+^/NADH ratio, lower malondialdehyde levels, and changes in genes related to energy metabolism and longevity [82,83]. These findings suggest that exogenous GABA can modulate oxidative-stress and energy-homeostasis biomarkers in this invertebrate model.

**Figure 4:**
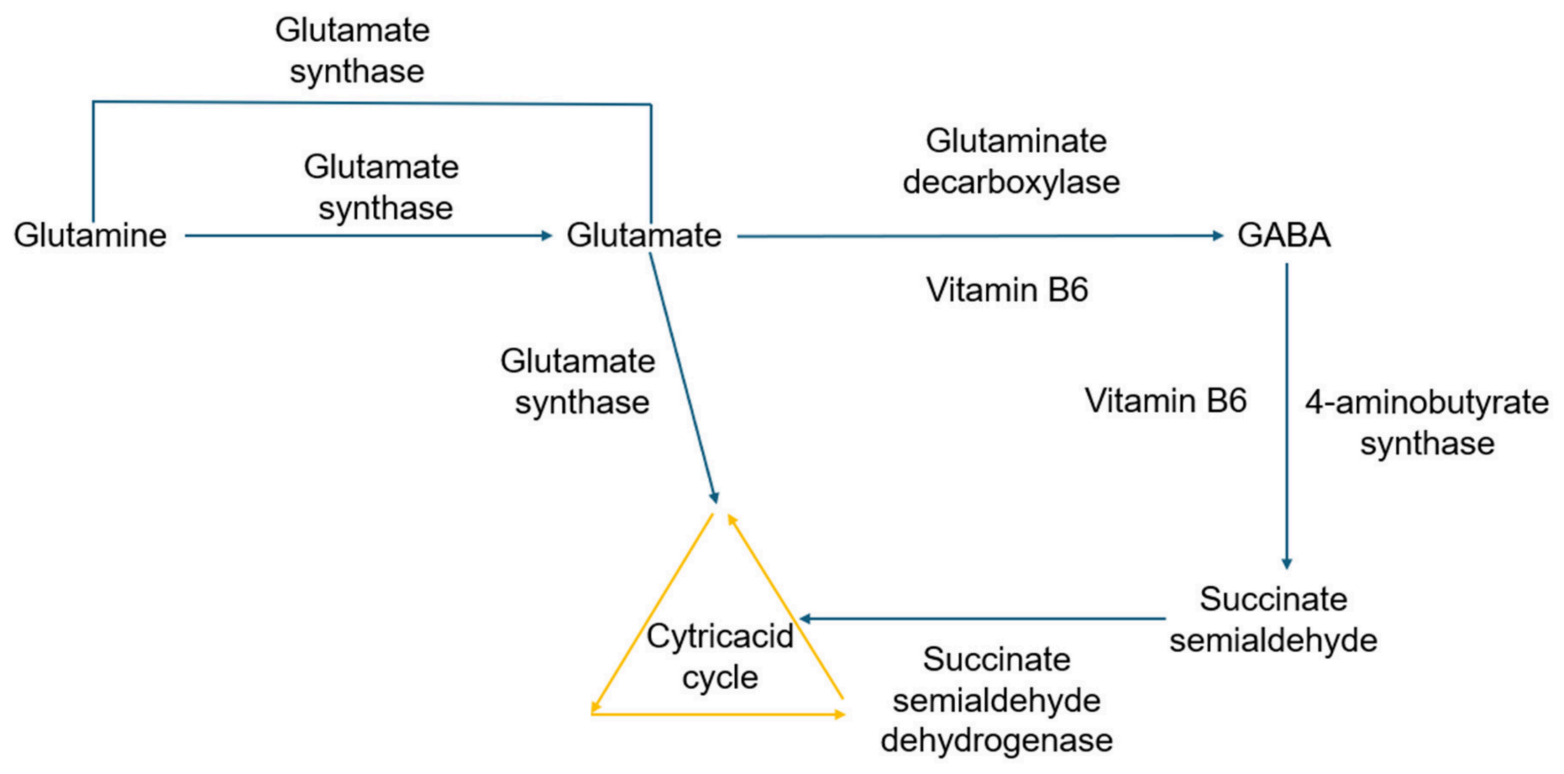
The metabolic pathway of the transformation of glutamate to GABA via the glutamate decarboxylase enzyme and vitamin B6.

Aging is associated with the accumulation of reactive oxygen species (ROS), which can cause cellular damage. Superoxide dismutase (SOD) and catalase (CAT) regulate the redox balance of the organism: SOD catalyzes the disproportionation of superoxide anion radicals (O_2_^−^) to H_2_O_2_ and O_2_ [84], while CAT decomposes H_2_O_2_ to water and O_2_, preventing H_2_O_2_-induced cellular toxicity [85]. As shown in Figure 4, GABA significantly increased SOD and CAT activities in nematodes. Compared with the control group, SOD and CAT activities in the 100 μg/mL GABA-treated group were increased by 52.46% and 52.52%, respectively.

Beyond the observed biomarker changes, the source and form of GABA warrant consideration. The silkworm study used purified GABA applied to feed under controlled conditions [82]; it did not evaluate GABA-rich human foods (i.e., complex food matrices) or determine the absorbed dose, tissue distribution, or bioavailability in such matrices. Gastrointestinal metabolism and interactions with other food components may further modify GABA exposure following ingestion. Accordingly, the available findings support the hypothesis that GABA may influence oxidative-stress-related and metabolic biomarkers in selected invertebrate models. These findings provide a rationale for mammalian and human research.

### 4.2. Molecular Mechanisms Underlying GABA’s Anti-Aging Effects

Aging of the human body mostly starts at the cellular level, with cells becoming damaged and undergoing apoptosis. Major theories of cellular damage include the free radical theory, telomere theory, and non-enzymatic glycosylation theory. The free radical theory suggests that excessive levels of free radicals—oxidants produced by normal cellular metabolism—can damage biomolecules and tissues, thereby accelerating the aging process [86]. Free radicals are characterized by the presence of one or more unpaired electrons and include reactive oxygen species (ROS), such as superoxide anion (O_2_^−^) and hydroxyl radical (·OH), as well as reactive nitrogen species (RNS), such as nitric oxide (NO) and nitrogen dioxide (NO_2_) [87,88]. When the balance between antioxidants and oxidants is disrupted, excessive free radical accumulation can damage the structure of nucleic acids, proteins, and lipids. Such damage to tissues and cells is associated with the development of various diseases, including diabetes, neurodegenerative disorders, cardiovascular disease, and rheumatoid arthritis [89,90].

Therefore, consuming natural products with antioxidant effects can help prevent free radical-induced diseases [89,91]. Numerous studies have indicated that GABA enhances the activities of antioxidant enzymes such as superoxide dismutase, glutathione peroxidase, and catalase, while also inhibiting oxidative stress markers including malondialdehyde and other reactive species [92]. For example, GABA has been reported to capture lipid peroxidation intermediates and reduce malondialdehyde levels, thereby potentially mitigating the inhibitory effects of reactive carbonyls on cellular energy metabolism. In a rat model of acute status epilepticus, GABA administration increased superoxide dismutase (SOD) and glutathione peroxidase (GSH-Px) activities in the mid-cortex and hippocampus, scavenging free radicals and protecting tissues from reactive carbonyls [93]. Studies using pancreatic cell lines have shown that GABA can induce human islet cells to secrete Klotho, an anti-aging protein that inhibits the generation of reactive oxygen species (ROS), reduces H_2_O_2_-induced oxidative stress, and decreases apoptosis [94,95]. This results in an enhanced antioxidant defense system, prolonged lifespan, and slowed age-related cognitive decline. In addition, GABA attenuated brain oxidative damage in streptozotocin-treated diabetic rats [96,97].

## 5. Alleviation of Menopausal Syndrome by GABA

### 5.1. Clinical Efficacy in Ameliorating Vasomotor and Psychological Symptoms

Nearly 75% of menopausal women experience distressing vasomotor symptoms, including hot flashes and night sweats [98]. GABA may modulate autonomic nervous system activity and influence hypothalamic thermoregulatory pathways, potentially through interactions with Ca^2+^ channels [99]. In an experimental analysis of 3500 menopausal women suffering from vasomotor symptoms, Shan et al. found that GABA administration led to a reduction in the frequency of hot flashes at both the 4th and 12th weeks [100]. In the hot flash control trial by Yoon et al., on the one hand, GABA was set up with a placebo dose range of 300 to 1800 mg/day. On the other hand, the variables were set as a composite score of hot flash frequency, duration, and hot flash severity, which were designed as a reference for comparing the effects of GABA versus placebo in the treatment of hot flashes. The study indicated a statistically significant reduction across the sets of experiments [101]. GABA and placebo doses ranged from 300 to 1800 mg/day. However, the meta-regression analysis revealed that GABA has predictive value in the frequency and severity of menopausal hot flashes [102] (i.e., to reflect the efficacy of treatment in alleviating menopausal hot flash symptoms, whether there are sequelae, etc.). But it does not affect the duration of hot flashes. In addition, 59 postmenopausal women with more than seven hot flashes per day were randomized to 900 mg/day GABA or placebo for 12 consecutive weeks. The study found that women treated with GABA had a 45% reduction in the frequency of hot flashes and a 54% reduction in the composite score.

### 5.2. Neuroendocrine Mechanisms Linking GABA to Menopausal Symptomatology

The onset of menopause is often accompanied by ovarian decline and reduced hypothalamic–pituitary–ovarian (HPO) axis activity, along with altered gonadotropin levels (elevated FSH and LH), collectively leading to reduced secretion of estradiol, progesterone, and their metabolites—including the neuroactive steroid allopregnanolone (ALLO), which acts as a positive allosteric modulator of GABA-A receptors. Changes in estradiol, progesterone, and their metabolites affect GABAergic neurons, potentially contributing to cognitive decline, low mood, depression, and memory impairment.

Studies conducted in premenopausal and postmenopausal women have found that GABA concentrations in the cerebral cortex are lower in menopausal women, particularly in those with depression [103]. Postmenopausal women with depression had significantly lower GABA levels in the anterior cingulate cortex/medial prefrontal cortex (ACC/mPFC) region. This reduction may impair physiological functions of the prefrontal cortex and ACC. This finding suggests that GABAergic neurons may be dysfunctional in postmenopausal women [104]. Electroconvulsive therapy, which can normalize brain GABA levels, has been observed to alleviate depression, suggesting a potential association between lower GABA levels and the pathogenesis of depression in postmenopausal women. On this basis, it has been speculated that appropriate GABA supplementation might improve age-related decline in GABAergic neuron function and alleviate menopausal mood symptoms. However, only a few reports have assessed GABAergic neuronal activity in postmenopausal women, and further research is needed.

## 6. The Anxiolytic Function of Dietary GABA

### 6.1. Psychophysiological Effects of Exogenous GABA Intake

#### 6.1.1. Modulation of Stress-Related Biomarkers by GABA-Rich Rice Bran Extract in Chronic Mild Stress Models

In a chronic mild stress (CMS) mouse model, Jeong et al. [105] investigated the effects of GABA-rich rice bran extract (GRBe) administered at doses of 500–2000 mg over four weeks. Compared to the CMS-control group, GRBe-fed mice showed altered levels of dopamine (DA), corticosterone (CS), 5-hydroxytryptamine (5-HT), and adrenocorticotropic hormone (ACTH) in both blood and brain tissues [105,106]. Additionally, GRBe administration reduced the expression of pro-inflammatory cytokines—interleukins (IL-1β, IL-2) and tumor necrosis factor (TNF-α)—which were elevated in the CMS-control group. Notably, IL-1β and IL-2 were significantly reduced in the GABA 1000 and GABA 2000 groups, and TNF-α was also decreased in GABA-treated groups [105,107].

#### 6.1.2. Electroencephalographic Correlates of GABA-Induced Relaxation

Human electroencephalography (EEG) studies offer a direct, non-invasive method to assess acute central nervous system responses to GABA intake. One frequently cited investigation examined the effects of a single 100 mg dose of GABA on brainwave activity in a small human cohort [108]. The study design involved dividing subjects into three groups: a control group (water only), an L-theanine group, and a GABA group (both receiving 100 mg of the respective compound). The key finding was that the GABA group showed a significant increase in the α-wave/β-wave ratio compared to the other two groups—a change conventionally interpreted as indicating a more relaxed mental state, given that α-waves are associated with wakeful relaxation while β-waves predominate during active concentration or stress [109]. With these constraints in mind, the EEG data provide exploratory human evidence that a single oral dose of GABA (100 mg) may influence cortical electrical activity in a pattern consistent with reduced mental stress. This finding is scientifically interesting and mechanistically plausible, but it should be described as an acute relaxation-associated electrophysiological response rather than as definitive evidence that “GABA resists anxiety”

#### 6.1.3. Immunomodulatory Influence of GABA on Salivary Ig A

Anxiety and sustained psychological stress have been associated with reduced secretory immunoglobulin A (sIgA) levels in saliva, making sIgA a commonly used non-invasive marker of mucosal immune function and stress status [110]. In a human experimental paradigm involving patients with a fear of heights crossing a suspension bridge, a placebo group showed a significant decrease in salivary IgA levels, whereas the GABA group initially exhibited a reduction in IgA that subsequently returned to baseline levels—remaining higher than the placebo group throughout the trial period [111].

This finding suggests that GABA intake may modulate stress-induced immune suppression, at least in the context of acute psychological stress provoked by a phobic stimulus. However, sIgA is an indirect biomarker with considerable intra- and inter-individual variability; changes in sIgA levels reflect systemic stress responses but do not independently validate anxiolytic efficacy. We interpret this as supportive but circumstantial evidence, complementary to the EEG findings, that GABA may have a modulating effect on stress-related physiological parameters. It should not be weighted as equivalent to clinical outcome measures such as validated anxiety scale scores (e.g., Hamilton Anxiety Rating Scale, State-Trait Anxiety Inventory), which are notably absent from the current literature on acute GABA supplementation.

### 6.2. Neurobiological Mechanisms of GABA in Anxiety Regulation

The neurobiological framework linking GABA to anxiety regulation is primarily derived from rodent neuropharmacology, neuroendocrine challenge studies, and receptor pharmacology. This body of research has generated a coherent mechanistic model that is widely cited in the literature—but its direct applicability to oral GABA intake in humans requires careful qualification.

At the systems level, it has been observed that anxiety states in both rodents and humans are associated with disruptions in central GABAergic neurotransmission, and that plasma and cerebrospinal fluid GABA concentrations are often lower in individuals with anxiety disorders than in healthy controls. Moreover, electroconvulsive therapy—an intervention that can alleviate severe depression and anxiety—has been shown to normalize brain GABA levels, suggesting a correlation between GABAergic function and affective state [112]. These correlational observations are informative, but they do not establish causation, nor do they prove that exogenous oral GABA can correct such deficits.

**Figure 5:**
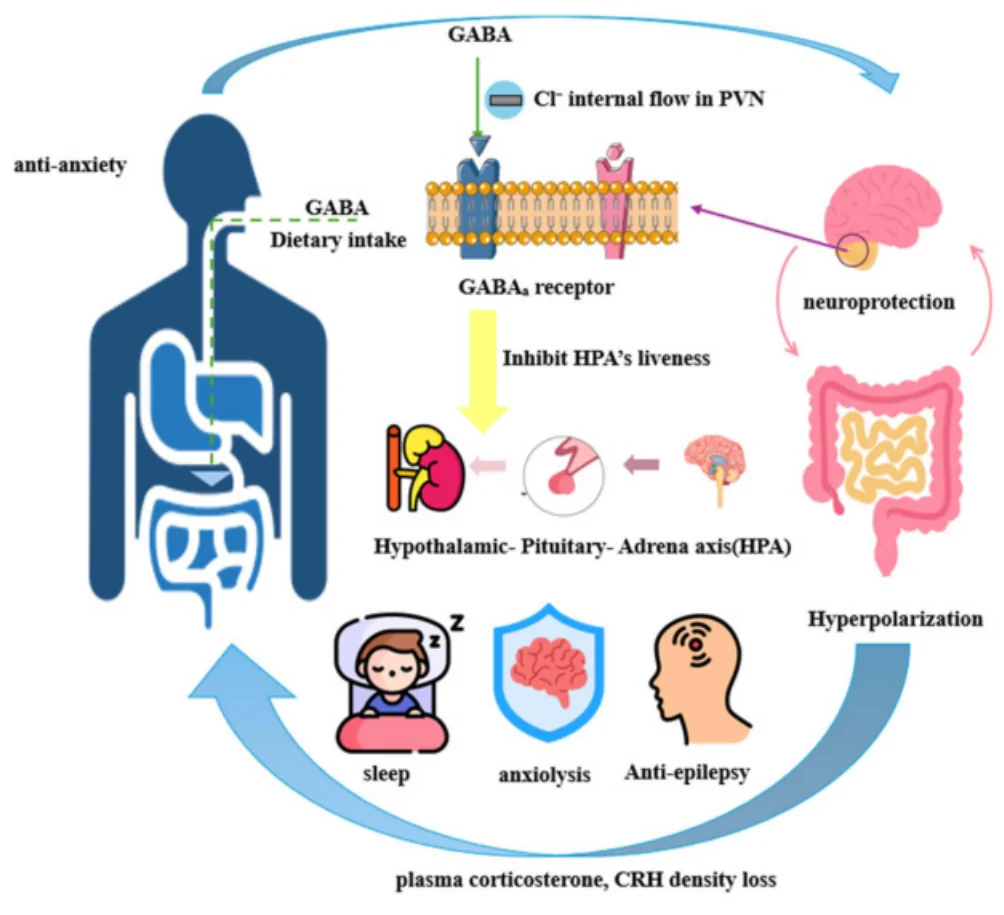
Neurobiological mechanisms of GABA in anxiety regulation: modulation of the HPA axis.

The dominant mechanistic hypothesis, illustrated in Figure 5, centers on the hypothalamic–pituitary–adrenal (HPA) axis—the body’s primary neuroendocrine stress-response system [113,114]. According to the rodent-based model, sustained stress stimulation impairs the inhibitory control of GABAergic neurons over corticotropin-releasing hormone (CRH)-producing neurons in the hypothalamus. This disinhibition leads to HPA axis hyperactivity, elevated plasma corticosterone (cortisol in humans), and consequent anxiety- or depression-like behaviors [65,115,116]. In this model, exogenous GABA is proposed to activate GABA_A_ receptors in the PVN, which inhibit HPA axis activity, prevent stress-induced rises in corticosterone and CRH, and thereby alleviate anxiety and depression [115].

We therefore suggest that the mechanistic discussion of GABA’s anxiolytic effects be framed as follows: preclinical (primarily rodent) studies have identified a plausible HPA axis-mediated GABAergic mechanism that merits further translational investigation. Human EEG and salivary biomarker studies provide preliminary, exploratory evidence that GABA intake may be associated with acute relaxation-related physiological changes. However, definitive evidence for clinically meaningful anxiolytic efficacy—particularly in diagnosed anxiety disorders—is currently lacking, and the mechanisms proposed in animal models should not be presented as established explanations for human effects. This calibrated interpretation aligns the review’s conclusions with the existing evidence base while identifying clear priorities for future clinical research (e.g., randomized controlled trials with validated psychiatric outcome measures, neuroimaging studies of brain GABA concentrations after oral dosing, and dose-finding studies to determine whether the proposed HPA axis modulation actually occurs in humans).

## 7. Sleep-Promoting Effects of GABA: Efficacy and Pathways

**Figure 6:**
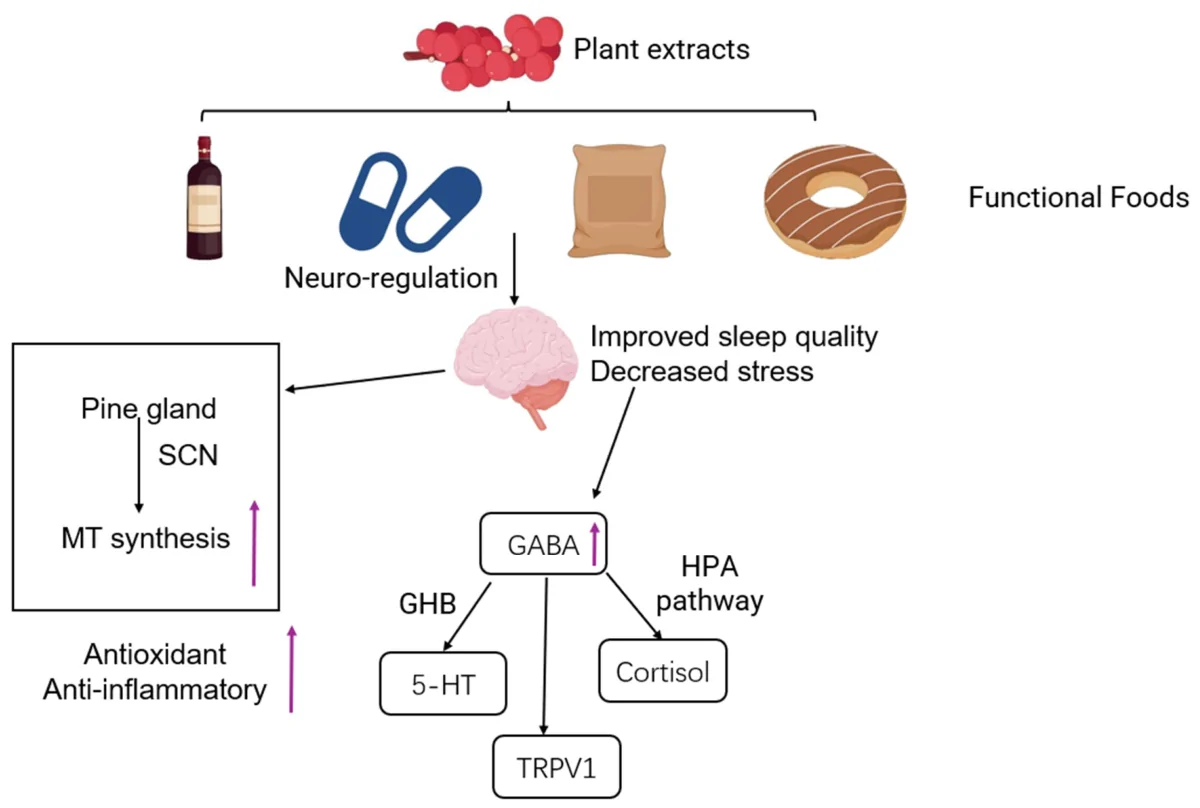
Neuro-regulation of sleep-associated plant extracts in skin health.

A good night‘s sleep is essential for relieving fatigue and maintaining overall health. Prolonged exposure to chronic stress can lead to hyperactivity of the hypothalamic–pituitary–adrenal (HPA) axis, which in turn may contribute to mood disturbances such as depression and anxiety, ultimately increasing the risk of insomnia. As a primary inhibitory neurotransmitter in the central nervous system, GABA promotes sleep primarily through direct activation of GABA_A_ receptors, which hyperpolarizes target neurons and reduces arousal-promoting neural activity (as discussed in Section 7.2). GABA is also catabolized via the GABA shunt pathway, with γ-hydroxybutyric acid (GHB) produced as a minor metabolite. However, this metabolic route is primarily a degradation pathway rather than the principal effector mechanism for GABA’s sleep-promoting effects [117]. As illustrated in Figure 6, GABA interacts with other neuromodulators—including melatonin and glutamate—within a complex network that collectively regulates the sleep–wake cycle in humans [118,119].

### 7.1. Improvements in Sleep Architecture Following GABA Supplementation

Yamatsu et al. [112] used electroencephalogram (EEG) to study the effects of GABA and placebo on sleep. A total of 10 subjects were randomly divided into two groups to take GABA and placebo in two drug administration periods. During the first dosing period, subjects in groups 1 and 2 were given 100 mg of GABA and a placebo 30 min before bedtime, and their EEG data were recorded for the first week.

A probability value of less than 5% is generally considered significant based on statistical significance. Observation of EEG shows that the effect of improving sleep after oral GABA is mainly reflected in the sleep onset and non-REM sleep periods [120]. GABA significantly shortened the sleep onset period by 5.0 min (the sleep onset period averaged about 10 min before GABA administration and was reduced by half after administration). There was no significant difference in the deep non-REM sleep latency, but the deep non-REM sleep latency was also shortened by 4.1 min in the GABA group [121]. GABA significantly increased the total duration of non-REM sleep by 2.2% (non-REM sleep is deep sleep, which is most beneficial for the brain and body to be rested, and a probability value of less than 5% is generally considered statistically significant).

### 7.2. Mechanism in GABA-Induced Sleep Modulation

GABA is thought to exert its sleep-improving effects through the gut–brain axis and the central nervous system. After oral administration, GABA is absorbed through the intestinal endothelial barrier (e.g., duodenum) via GABA transporter proteins—a process that forms part of the gut–brain axis, a bidirectional information exchange system between the intestine and the brain. GABA receptors in the human brain contain binding sites that can selectively inhibit neurons involved in sleep promotion [115,122], For example, the α-subunit of GABA_A_ receptors contains the binding site for benzodiazepine sedative-hypnotic drugs. Hong et al. demonstrated that GABA and 5-hydroxytryptophan (5-HTP) significantly shortened sleep latency and prolonged total sleep time in mice after gavage [123]. Real-time fluorescence quantitative PCR analysis showed that the transcript level of GABA_A_ receptors in the cerebral cortex of treated mice was 1.37 times higher than that of the control group [124]. These findings suggest that exogenous GABA can increase both GABA content and GABA_A_ receptor expression in the central nervous system of animal models, potentially contributing to sleep promotion.

Between brain tissue and circulating blood lies the blood–brain barrier (BBB), a dynamic barrier composed of capillary endothelial cells and astrocytes that protects the central nervous system from toxic substances by restricting the passage of hydrophilic molecules [125]. Given the restrictive nature of the BBB, it is important to distinguish between different routes of administration and experimental models when evaluating GABA’s central effects. Early pharmacokinetic assumptions held that GABA, being weakly lipophilic, could not efficiently cross the BBB from the systemic circulation. However, in a rat study using intravenous (not oral) administration, Li et al. reported that 60 min after a 50 mg/kg intravenous injection, GABA concentrations in brain tissue reached 474 ± 33 μg/g—significantly higher than those in saline-injected controls—and the animals exhibited sedative-like behaviors [126]. The authors attributed this to the presence of the BGT-1 transporter protein in cerebrovascular endothelial cells, which may facilitate GABA uptake into the brain [127].

Therefore, while the BGT-1-mediated transport mechanism offers a plausible molecular basis for BBB permeability, its relevance to orally administered dietary GABA in humans remains unconfirmed. Future studies employing oral administration in larger animal models, combined with cerebrospinal fluid or microdialysis sampling, are needed to bridge this translational gap [128]. For the purposes of this review, the sleep-promoting effects discussed in human studies may involve both central (if BBB penetration occurs) and peripheral (e.g., vagal or enteric) mechanisms, and the relative contribution of each remains an open question.

## 8. Anticancer Role of GABA: Cellular and Molecular Perspectives

GABA has been shown to regulate the proliferation, migration, and metastasis of tumor cells in vitro by binding to its receptors and modulating specific signaling pathways. Its proposed anticancer activities in cell-based models include three main mechanisms: (1) inhibiting cell proliferation and migration by reducing cyclic adenosine monophosphate (cAMP) production in tumor cells; (2) suppressing matrix metalloproteinase (MMP) production; and (3) inhibiting tumor angiogenesis by reducing the production of vascular endothelial growth factor (VEGF) and prostaglandin E2 (PGE2) [129]. The effects of GABA on cancer cells appear to be highly dependent on tumor cell type, intracellular signaling context, and receptor subtype expression.

### 8.1. The Therapeutic Effect of GABA on Anticancer

Taking colorectal cancer cells as an example, a 100 μM concentration of GABA was used to act on colorectal cancer cells SW620, SW480, and HCT116 cells (a 100 μM concentration of GABA was most effective in inhibiting proliferation), and it was found that the cell cycles of the three kinds of cells were altered [130]. Among them, the number of S-phase cells of SW620 and SW480 decreased by 15% and 13%. The reduction in S-phase cells indicated that DNA synthesis was blocked, and the growth of the two cell lines was inhibited. For HCT116 cells, the number of G2/M phases increased by 30% after GABA treatment, and the G2/M phase is the late stage of DNA synthesis and division. Hence, the number of G1/G0-phase cells in the next division cycle decreased (G1/G0 phase is the period to prepare material and energy for the DNA replication in the next stage of S phase), which led to a delay in the division of HCT116 cells. Therefore, GABA interferes with the proliferation of cancer cells by interfering with their cell division cycle.

### 8.2. Mechanism of GABA Inhibition on Different Cancers

For hepatocellular carcinoma cells, GABA prevents metastasis and invasion of hepatocellular carcinoma cells by binding to GABA_A_ receptors to induce cytoskeletal reorganization of hepatocellular carcinoma cells or by activating GABA_A_ receptors to inhibit the expression of mRNA for α-fetoprotein (a protein abnormally expressed in cancer cells) [131]; GABA mediates GABA_B_ receptors to inhibit the cAMP signaling pathway that thereby blocking pancreatic cancer progression in pancreatic cancer cells [132]; GABA significantly down-regulated the mRNA expression of genes such as FOS in colorectal cancer cells. In breast cancer tumor cells, FOS and FOSB function as oncogenes, and when GABA silences the FOS gene, it can block the synthesis of c-fos proteins in the cell membrane, leading to the blockage of phospholipid synthesis in the cell membrane and the inhibition of cancer cell proliferation, GABA_B_ receptors bind to β-inhibitory proteins to inhibit breast cancer cell migration and induce apoptosis [133,134].

## 9. Antihyperglycemic Role of GABA

### 9.1. Mechanisms of GABA in Regulating Insulin and Glucagon Secretion

**Figure 7:**
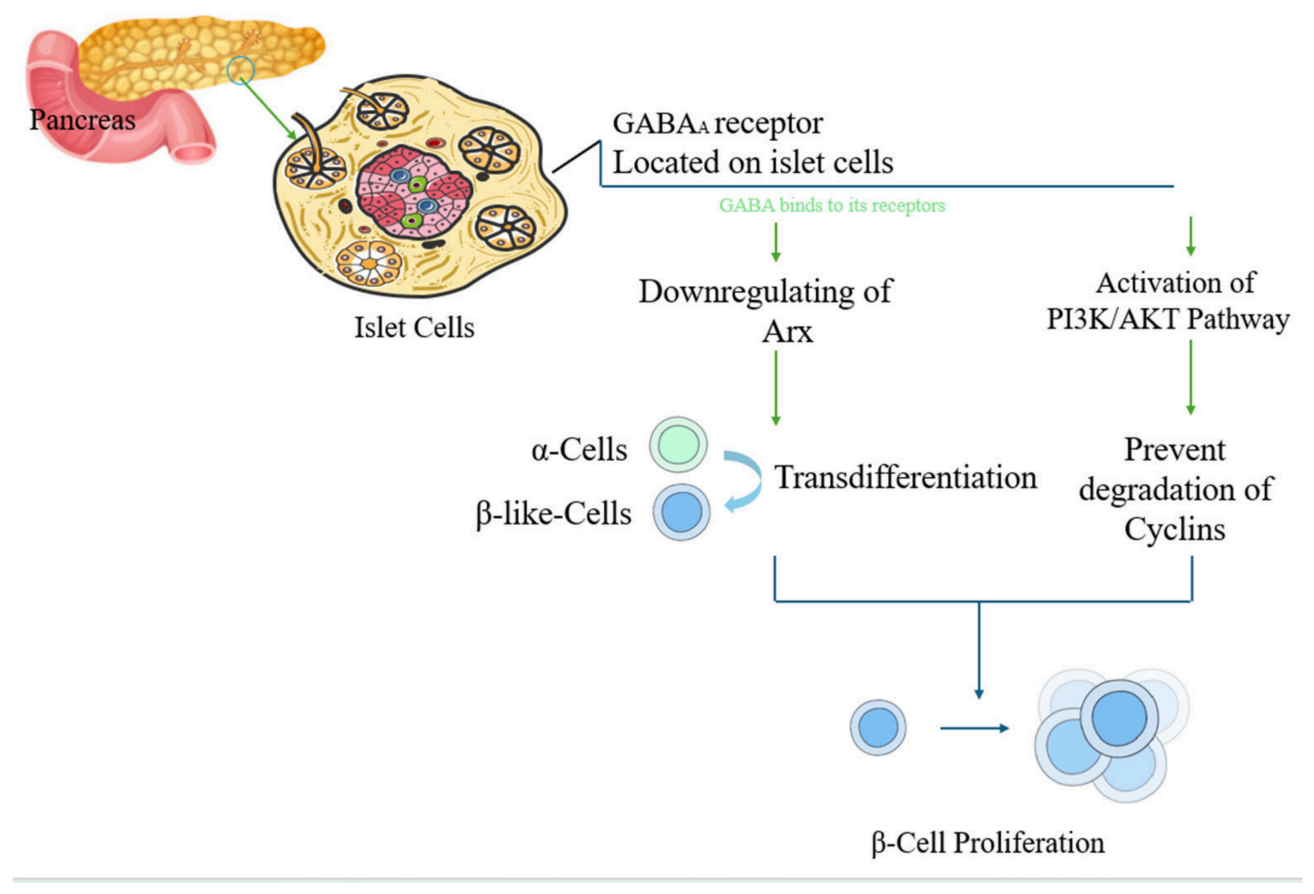
GABA’s mechanism of action in promoting Panrea β cell proliferation.

Under normal physiological conditions, hyperglycemia stimulates β-cells to secrete insulin while simultaneously inhibiting α-cell glucagon secretion—a coordinated response that maintains blood glucose levels within the physiological range. At the cellular electrophysiological level, the actions of GABA on α- and β-cells are mediated through distinct membrane potential effects. In pancreatic α-cells, GABA activates GABA-A receptors, leading to chloride influx, membrane hyperpolarization, and inhibition of glucagon secretion [135]. As shown in Figure 7, in pancreatic β-cells, by contrast, the same neurotransmitter has been reported to depolarize the membrane potential, thereby enhancing calcium influx and promoting insulin exocytosis [136]. This dual action—suppressing the hyperglycemic hormone while stimulating the hypoglycemic hormone—makes GABA a functionally attractive candidate for blood glucose regulation.

Further mechanistic studies have revealed intracellular signaling components that may underlie these effects. After GABA administration, Akt is rapidly activated, leading to phosphorylation of GABA-A receptors and their translocation to the cell membrane surface. This trafficking event enhances the β-cell’s responsiveness to subsequent GABA stimulation, potentially reinforcing insulin secretion and contributing to glucose homeostasis [136,137].

### 9.2. Current Status in Diabetes Management

Type 1 and type 2 diabetes share the common feature of progressive β-cell dysfunction or loss, making the preservation and regeneration of remaining β-cells an attractive therapeutic target. In a series of preclinical studies using isolated pancreatic islets and rodent models, Tian et al. demonstrated that GABA can modulate insulin and glucagon secretion, protect β-cells from apoptosis, and promote β-cell replication under certain experimental conditions [138,139,140]. Additionally, GABA has been shown to attenuate immune activation and inflammation in diabetic mouse models, thereby contributing to improved glucose homeostasis [141]. In a study of non-obese diabetic (NOD) mice—a well-established animal model of autoimmune type 1 diabetes—combined therapy with GABA and proinsulin/alum was reported to maintain normoglycemia in newly diabetic NOD mice over an extended period, whereas neither agent alone was effective. The proposed mechanism involved suppression of pathogenic T-cell responses, induction of regulatory T-cell responses, and promotion of β-cell proliferation [141,142].

Therefore, the statement that “GABA therapy is promising in treating diabetes” should be understood in the context of preclinical research, not as an established clinical indication. The NOD mouse data provide a compelling rationale for future translational investigations—specifically, pilot phase I/II trials in humans to assess safety, pharmacokinetics, and preliminary efficacy in recently diagnosed type 1 diabetes patients—but they do not constitute proof of therapeutic efficacy in humans [138]. We emphasize this distinction to align the review’s conclusions with the current evidence base and to identify this area as a high-priority direction for future research.

## 10. Amount of GABA Pharmacological Action and Safety

### 10.1. Appropriate Amount of Pharmacological Effect

As of April 2021, 644 products in the U.S. Dietary Supplement Labeling Database (DSLD) contained GABA, primarily labeled for adults aged 18 years and older [143]. Manufacturers’ recommended intakes range from 1.5 µg (0.0015 mg) to 3000 mg/day, with the most common recommendation being approximately 100 mg/day, typically divided into three doses [144].

At acute single doses and typical recommended doses (≤300 mg/day), available human data indicate that GABA is generally well-tolerated. In a 12-week randomized controlled trial involving 86 hypertensive subjects who consumed fermented milk containing 10 mg GABA daily, no significant differences were observed between the GABA and placebo groups across a wide range of safety parameters, including body weight, body mass index, heart rate, urine indices, standard blood clinical chemistry, and hematology [145]. Similarly, a 32-day rat feeding study (discussed in Section 10.2 below) reported no morbidity, mortality, or organ-weight changes outside the historical control range.

In contrast, GABA is classified differently across regulatory jurisdictions. In Canada, GABA-containing products are regulated as Natural Health Products; Health Canada has licensed 119 such products in its Natural Health Products Database as of April 2021, with a recommended daily intake of 50–3000 mg and a single-dose limit of 750 mg. In the European Union and Australia, GABA is subject to food or therapeutic goods regulations depending on product presentation and health claims. However, other Western countries do not specify a standardized recommended intake for GABA. The 18 g/day dose is 60 times higher than the typical 300 mg/day supplement and 36 times higher than the Chinese national standard of 500 mg/day. Although adverse effects were observed at this pharmacological dose, this does not necessarily indicate that lower, food-relevant doses carry similar risks. Nevertheless, it does establish that GABA exerts physiological effects at very high intake levels, particularly on the endocrine system (growth hormone and prolactin modulation) [146]. The reported throat burning and dyspnea at 18 g/day, although transient, should be explicitly acknowledged in any safety summary rather than omitted or dismissed.

For repeated use beyond four weeks or at doses exceeding 300 mg/day, current guidance advises medical consultation [145]. This recommendation reflects the absence of long-term (>6 months) human safety data at higher doses, rather than the presence of established toxicity. The key evidence gap is not acute toxicity—which appears minimal—but the lack of chronic exposure data in diverse populations. Pregnant and lactating women are specifically advised to use GABA with caution, as GABA influences neurotransmitter and endocrine systems, including increases in growth hormone and prolactin under certain experimental conditions [147].

### 10.2. Safety and Precautions

Animal toxicology studies provide supporting evidence for the safety of GABA at dietary-relevant intake levels. In a representative study by the Japan Science and Food Association, 32-day-old rats were fed a diet containing 1% GABA or a standard control diet. The only statistically significant difference between groups was a 7% (control) and 6% (GABA-fed) increase in testis weight—both within the historical control range for this strain—and no changes were observed in blood biochemical indices, organ weights (other than the testis finding), body weight, food intake, or morbidity/mortality [73]. In isolation, a 6–7% increase in testis weight within the historical control range, unaccompanied by histopathological changes or functional impairment, is generally considered biologically insignificant in toxicological risk assessment. The absence of a dose–response relationship, the lack of corroborating histopathology, and the fact that both control and treatment groups fell within the same historical reference range collectively suggest that this is a random or incidental finding rather than a treatment-related effect [148]. It should not be highlighted as a “significant difference” in a manner that implies a safety concern. Rather, the overall conclusion from this study is that no treatment-related adverse effects were identified at the tested dietary inclusion level.

However, it must be acknowledged that the 1% GABA diet in rats represents an exposure level substantially higher than human dietary intake on a mg/kg basis (species scaling considerations apply) [147], and the study duration (32 days) is subchronic rather than chronic. Long-term carcinogenicity and reproductive toxicology studies are not available in the public literature reviewed here [149].

## 11. Conclusions and Perspectives

In summary, GABA is a water-soluble, non-protein amino acid with favorable physicochemical stability. In preclinical models and preliminary human studies, it has been associated with effects on sleep architecture, stress-related physiological markers, blood pressure, and glycemic parameters, although the strength of evidence varies considerably across these endpoints. The direct translation of these findings to clinical recommendations requires further investigation. Available safety data, including those reviewed by the United States Pharmacopeia for specific ingredient monographs, indicate that GABA is generally well-tolerated at typical dietary supplement doses. However, as discussed in Section 10, the evidence base for long-term safety, high-dose use (≥3000 mg/day), and use in special populations remains limited. Given its broad physiological benefits and exemplary safety profile, GABA-enriched functional foods have gained widespread popularity among consumers.

Looking ahead, the future of GABA research will likely focus on optimizing GABA-enriched functional foods tailored to specific populations, such as children, the elderly, and individuals with chronic conditions like hypertension and diabetes. The continued advancement of microbial fermentation and crop fortification technologies will play a crucial role in improving the scalability and cost-effectiveness of GABA production, making these products more widely accessible. Moreover, further studies will be essential to evaluate the safety and efficacy of higher-dose GABA supplementation, particularly regarding its long-term effects on diverse age groups and individuals with different health conditions.

Personalized nutrition is another promising direction, with potential breakthroughs in genomic medicine allowing for tailored GABA intake recommendations based on individual genetic profiles and metabolic needs. Investigating GABA’s role in neuromodulation, cognitive function enhancement, and mental health treatment may also open up new therapeutic possibilities. With the growing consumer interest in functional foods and the preliminary evidence supporting GABA’s bioactivity in several physiological domains, continued research—particularly well-controlled human trials with dose-ranging designs and long-term follow-up—will be essential to determine whether and how GABA-enriched foods can contribute to public health and well-being.

## Figures and Tables

**Table 1:** Natural gamma-aminobutyric acid content in daily foods [22].

	Species	The Content of GABA	References
Cereals	Brown rice	5.28–27 mg/100 g	[23,24,25,26]
	Red rice	1.18–2.91 mg/100 g	[27,28]
	Black rice	0.67–7.46 mg/100 g	[29]
	Wheat	4.55–14.68 mg/100 g	[30,31,32]
	Barley	1.96–54.00 mg/100 g	[33,34,35]
	Corn	15.27 mg/100 g	[36]
Beans	Yellow soybean	12.00–19.00 mg/100 g	[37,38]
	Black soybean	4.38–61.00 mg/100 g	[38,39]
	Lupin	46.00 mg/100 g	[40]
	Faba bean	5.00 mg/100 g	[41]
	Chickpea	6.42 mg/100 g	[42]
Pseudo-cereals	Quinoa	7.00–66.10 mg/100 g	[11]
	Common buckwheat	10.34 mg/100 g	[43]
	Tartary buckwheat	42.60–112.50 mg/100 g	[44]
Fruits	Grape	5.89–10.98 mg/100 g	[45]
	Apple	9.72 mg/100 g	[46]
	Mulberry fruit	17.10–33.60 mg/100 g	[47]
	Kiwifruit	2.54–19.14 mg/100 g	[48]
	Strawberry	1.55–3.61 mg/100 g	[49]
	Lychee	170.00–350.00 mg/100 g	[50]
Vegetables	Tomato	219.86–404.89 mg/100 g	[51]
	Spinach	232.10–381.00 mg/100 g	[52]
	Sweet potato	1.38–5.18 mg/100 g	[53]
	Fresh cucumber	8.54 mg/100 g	[54]
	Eggplant	23.28–38.12 mg/100 g	[55]
Tea	White tea	3.49–207.00 mg/100 g	[56,57]
	Olong tea	1.86–97.00 mg/100 g	[57,58]
	Green tea	0.24–87.00 mg/100 g	
	Black tea	2.65–55.50 mg/100 g	
	Pu’er tea	0–4.61 mg/100 g	[58,59]
Edible mushrooms	White mushroom	18.00–20.00 mg/100 g	[60]
	Shiitake mushroom	17.00–35.00 mg/100 g	[61]
	Oyster mushroom	32.15–57.73 mg/100 g	[62]
Others	Chestnut	1.03–1.65 mg/100 g	[63]
	Cocoa beans	31.70–101.20 mg/100 g	[56,64]

## Data Availability

No new data were created or analyzed in this study. Data sharing is not applicable to this article.
